# The Impact of Online Pandemic-Related Information on Prosocial Behavior among Healthcare Students: The Role of Emotional Contagion and Epistemic Motivation

**DOI:** 10.3390/bs14100945

**Published:** 2024-10-14

**Authors:** Shiyu Zhou, Jing Chang, Yang Yang, Yue Han, Chang Liu, Yuchen Jiao, Yao Meng, Yan Ji

**Affiliations:** 1 School of Nursing, Nanjing Medical University, 101 Longmian Avenue, Jiangning District, Nanjing 211166, China; zsy20000927@163.com (S.Z.); cjzmfs@163.com (J.C.); yangyang20212204@163.com (Y.Y.); 2022121350@stu.njmu.edu.cn (Y.H.); 2 The Health Supervision Institute of Zhenjiang, 9 Huangshan South Road, Runzhou District, Zhenjiang 212002, China; walnut1997@163.com; 3 Department of Radiotherapy, The Affiliated Cancer Hospital of Nanjing Medical University, 42 Baiziting Road, Xuanwu District, Nanjing 210009, China; jyc12342021@163.com

**Keywords:** pandemic information, healthcare students, prosocial behaviors, emotional contagion, epistemic motivation

## Abstract

Prosocial behavior is fundamental for healthcare students, shaped by their traits and the external environment. Online information seeking is the most commonly used way for healthcare students to access pandemics; however, it is unclear whether the willingness of healthcare students to help others will be affected by pandemic information via the Internet environment. The current study takes the COVID-19 pandemic as an example, investigating how 81 healthcare students with varying prosocial tendencies behaved helpfully after being exposed to pandemic-related pictures online. Study 1 measured the influence of emotional contagion (positive emotion/negative emotion) from online information on students’ prosocial behavior; Study 2 examined online information’s influence on prosocial behavior by controlling individuals’ epistemic motivation (goal-directed task/no-goal-directed task) to gain pandemic information. The results indicated that negative pandemic information was more likely to influence students with low prosocial tendencies, which would then lead to a decrease in prosocial actions (F = 7.842, *p* = 0.005). Further, students with low prosocial tendencies were more likely to engage in prosocial behavior when they did not aim goal-directed attention to the pandemic-related information, compared to those with goal-directed attention (F = 9.159, *p* = 0.003). Participants with high prosocial tendencies did not differ much in helping others (*p* > 0.05). The results indicated that only healthcare students with limited prosocial tendencies were less inclined to assist others after receiving negative information about the pandemic. Thus, reducing their deliberate approach to online information related to the pandemic will increase their chances of taking prosocial behavior.

## 1. Introduction

Prosocial behavior is essential for healthcare professionals and has the potential to improve healthcare effectiveness, especially in times of international public health emergencies [1,2]. Given this immense backdrop, concern over how the pandemic is affecting prosocial behavior among healthcare professionals is growing. In the case of COVID-19, the pandemic not only impacts the mental health of healthcare workers, but also hinders their ability to assist in the pandemic [2,3]. However, there have been mixed results regarding how the pandemic has affected healthcare students’ prosocial behavior. Research indicates that most students are willing to help in a medical capacity [4]; however, another study shows that less than 50% of the students were willing to volunteer for activities involving patients [5]. Findings suggested the need to look beyond the simplicity of altruism to the role of operational and motivational factors to explain students’ decision behind volunteer behavior [6]. Therefore, understanding the factors that influence the prosocial behavior of healthcare students (future healthcare providers) in the context of an epidemic outbreak is crucial for the future development of public health.

One of the primary factors influencing the incidence of individual prosocial behavior is the interpretation of the external environment. During a pandemic, healthcare students are more likely to acquire pandemic information from the Internet [7]. For example, research shows a link between access to COVID-19 information and higher distress levels among nursing students [8]. Around 40.9% of the healthcare students believed that the “COVID-19 infodemic” adversely affected their health, while 23.2% reported low mood, and 15.5% reported anxiety [9]. As a matter of fact, the pandemic has led to increased job insecurity, anxiety, and burnout among healthcare students, which may reduce their desire to help others [10,11]. Therefore, how pandemic-related information online affects the occurrence of healthcare students’ prosocial behavior needs to be further clarified.

According to the hypothesis of emotions as a social information model (EASI), emotions are the main source of social influence. Emotional contagion is one of the factors that affects individual information reception and has an impact on individual decision-making behavior [12,13,14]. There was also evidence that when someone is infected with a network emotion, these emotions can impact their decisions and judgments [15,16]. Further, neurophysiological studies showed that emotion affects individuals’ prosocial behavior performance [17]. Positive information stimuli are beneficial for individuals’ desire to perform prosocial behaviors, while negative information may do the opposite [18]. The thing is, sadness narrows individuals’ attention spans and makes them more self-focused, which may inhibit prosocial behaviors [19,20]. Research shows that when emotions like anxiety arise during the COVID-19 pandemic, healthcare students are typically less willing to engage in helping behaviors [21]. Pandemic-related information can have varying effects, as follows: negative information, like high infection risks and rising death tolls, may lead to egocentric behavior, while positive information, such as donations and volunteerism, can encourage empathy in students [22,23]. Thus, we induce that while receiving positive information about the pandemic boosted healthcare students’ desire to help others, receiving negative information about COVID-19 would have the opposite effect.

In addition to emotional contagion, motivation processing is another critical dimension of information reception. Previous research has primarily explored the causes of prosocial behavior from the perspectives of altruistic motivation [24] and social exchange motivation [25,26]. However, these motivations are stable patterns shaped by social and familial influence, and they are not enough to express the impact that sudden environmental changes (such as pandemics) have on individuals’ behaviors. Instead, EASI believes that individuals with epistemic motivation can intentionally focus deeply on outside information, affecting how they make decisions [13]. Thus, it follows that deliberate attention directed to online pandemic information may, likewise, impact peoples’ decisions to engage in prosocial behavior. Studies have also shown that if individuals reduce their attention directed to negative information, or change their cognition, they will be conducive to the generation of empathy [27,28]. Thus, it can be inferred that although healthcare students may struggle to avoid negative online information about the pandemic, they can lessen their deliberate attention to it. This may help mitigate the decline in prosocial behaviors driven by negative emotions.

**Figure 1:**
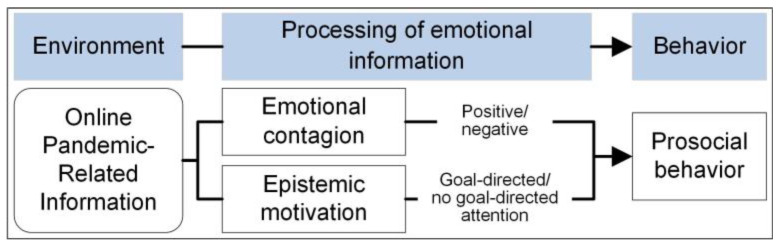
The research design. Based on the hypothesis of emotions as a social information model (EASI), prosocial behavior could be influenced by the processing of emotional contagion and epistemic motivation.

In addition to online information related to the pandemic, another factor influencing the incidence of prosocial behavior among healthcare students is their prosocial tendencies. It is important to note that individuals with a strong inclination to help others are less swayed by their surroundings, and their acts of kindness align more closely with their true feelings [22,29,30]. Thus, to investigate the occurrence of prosocial behavior in individuals, it is necessary to consider their prosocial characteristics. Overall, this study selected healthcare students with varying prosocial tendencies as participants and utilized online COVID-19 news pictures related to healthcare workers as study materials (Figure 1). Study 1 measured the influence of emotional contagion (positive/negative emotion) of online information on students’ prosocial behavior. Hypothesis 1 posited that negative pandemic information would inhibit healthcare students from engaging in prosocial behavior. Study 2 examined the influence on prosocial behavior by controlling individuals’ epistemic motivation (goal-directed task/no-goal-directed task) toward pandemic information. Hypothesis 2 suggested that healthcare students with low prosocial tendencies are less likely to engage in prosocial behaviors after being exposed to online pandemic information, while those with high prosocial tendencies show no change in their behavior.

## 2. Study 1

### 2.1. Participants

Participants were recruited from a local university by posting posters and sharing online questionnaires. Only one response per account, device, and IP address could be submitted. A total of 152 participants completed the baseline data survey containing demographic information and the Prosocial Tendency Measurement Scale. Of these, participants with prosocial tendency scores in the top 24% and bottom 24% were included in the subsequent experimental study, and they were divided into high and low prosocial groups, respectively [31]. These students were sent an individualized message invitation to participate in the study through QQ Instant Messenger. Inclusion criteria were as follows: (1) undergraduate healthcare students majoring in nursing, clinical medicine, and other faculties related to clinical practice; (2) right-handed; (3) no cognitive impairment, verbal communication impairment, or pre-existing emotional problems, trauma, etc. Exclusion criteria were as follows: (1) those who temporarily and voluntarily withdrew before the study or did not complete all tasks during the study; (2) failure to follow the requirements or to achieve the expected effects of stimuli in the study; (3) data loss. Ninety-one healthcare students were assigned to two prosocial tendency groups. Of these, seventeen participants were not included, which is attributed to the unaccomplished helping task of nine, the failed emotional arousal of seven, and the missing program data of one. At last, 34 participants with high prosocial tendencies and 40 participants with low prosocial tendencies were included in the analysis. All participants participated voluntarily and signed written informed consent. The Ethics Committee of Nanjing Medical University (2022-No.808) awarded its approval for this study.

### 2.2. Materials

#### 2.2.1. Prosocial Tendency

The Prosocial Tendencies Measurement Scale was used to measure healthcare students’ prosocial tendencies [32,33]. It contains 23 questions, asked with 5-point Likert responses ranging from “1 = absolutely inconsistent” to “5 = absolutely consistent”. An overall score was calculated, with the higher score indicating greater prosocial tendency. This scale was often used among college students. A sample item is the following: “I can help others best when people are watching me.” Cronbach’s α of this scale in the current research was 0.85.

#### 2.2.2. Positive/Negative Emotion

The Positive Affect and Negative Affect Scale (PANAS) [34] is divided into two dimensions, positive and negative emotions, each containing 10 descriptors asked with 5-point Likert responses to assess the subject’s feelings, reactions, and level of identification. Higher scores indicate higher positive or negative emotions. This study used the scale to investigate whether an individual’s emotional response was more positive or negative after exposure to relevant information in the task.

#### 2.2.3. Emotional Contagion Task

We selected actual social media pictures related to the pandemic for the experimental task materials of emotional contagion. Picture evaluation includes the following two aspects: arousal and valence [35]. Emotional valence ranges from positive to negative, indicating the response of the motivational system to a stimulus, while emotional arousal varies from calm to excited, reflecting the tension level triggered by the stimulus. Thus, emotional information in tasks includes valence (positive, neutral, negative) and arousal (high, low), which can evoke corresponding emotional responses in students to different types of information [36]. We first selected 72 COVID-19 news pictures from the Internet, and then recruited 20 healthcare students to rate the valence and arousal of each picture on a 7-point scale, with higher scores denoting greater positivity and arousal. At last, we chose 40 pictures from these pictures to provide visual stimuli for emotional contagion (20 positive, 20 negative).

**Figure 2:**
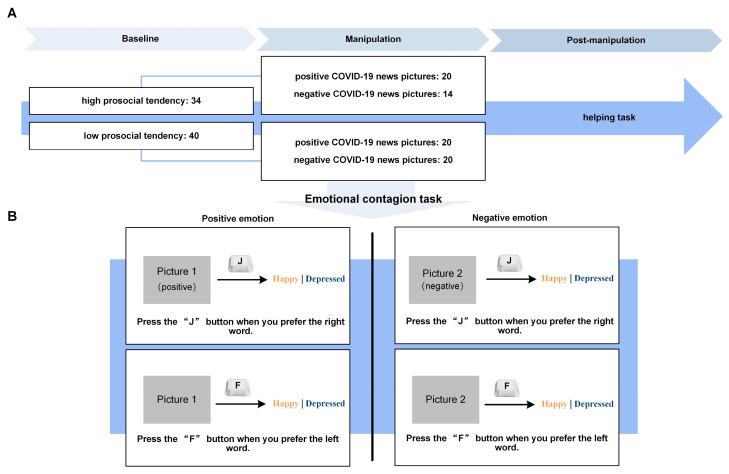
An Overview of the Procedure in Study 1. (**A**) Study 1 task path and (**B**) manipulations in the emotional contagion task. All visual stimuli were displayed using E-prime 2.0 on a Lenovo laptop with a 13-inch monitor. In the emotional contagion task, participants were presented with 20 of the critical photographs randomly (20 positive, 20 negative).

The emotional contagion task is realized using the classical emotional arousal paradigm [37]. There were 3 runs, 2 blocks per run, 20 trials per block, and a total of 120 trials. After the task instructions and practice, a fixation cross appeared for 1 s, followed by a randomly displayed picture for 3 s. Then, another fixation cross appeared for 1 s, and finally, two words representing positive and negative emotions were shown on the screen for the participant to choose by pressing a key. Participants were asked to press the “J” button when they preferred the right word, and to press the “F” button when they preferred the left word in the positive emotional task and negative emotional task (see Figure 2B). The participants’ reaction time from the appearance of the word to the pressing of the key was also recorded.

#### 2.2.4. Prosocial Behavior Task

Healthcare students’ prosocial behavior was assessed using an incentivized “helping task,” based on an incentivized decision-making task [38]. The task involves a hypothetical scenario where you will be randomly paired with another student in the experiment. If you are the strong participant (the decider who allocates funds), the other student will be the weak participant (the receiver). You both will remain anonymous to each other; the receiver will be informed of the decider’s choices, but will not know that you are the decider. How will you allocate the funds? In this task, participants decided whether to divide a certain outcome (15 CNY, 100 CNY, or 1000 CNY) equally between themselves and another participant. You need to choose between options A and B. Option A is to keep all of the funds for yourself, while B is to help the weaker person and share the funds equally with them; however, the former option requires a 20% deduction of the cost of the help. For example, if you choose A, you will receive 100% of the 15 yuan, and the recipient will receive 100% of the 0 yuan; if you choose B, you will receive 100% of the 6 yuan, and the recipient will receive 100% of the 6 yuan (minus the 3 yuan cost). Thus, the participant chooses between helping and not helping. We assigned a 0 value to the selfish option, and a 1 value to the prosocial option.

### 2.3. Procedure

Firstly, participants were required to complete an emotional contagion task that included emotional pictures related to the pandemic. Secondly, the Positive Affect and Negative Affect Scale (PANAS) was used to measure the students’ emotional state before and after the emotional contagion task. The success of emotional contagion was judged by comparing the students’ pre- and post-emotional states. Lastly, participants were asked to finish the prosocial behavior task after the emotional contagion task. The experiments were conducted uniformly in the laboratory, and all participants signed an informed consent form before the experiment. A total of 30 CNY was given to each participant after the experiment.

### 2.4. Data Analysis

The study was modeled by a 2 (high prosocial tendency/low prosocial tendency) × 2 (positive/negative pictures) mixed measure analysis of variance (ANOVA) (presented in Figure 2A). Prosocial behavior decision data were used as the dependent variable. According to the baseline prosocial tendency measurement, 74 participants were divided into a high prosocial tendency group (34 healthcare students) and a low prosocial tendency group (40 healthcare students). They were then randomly assigned to the positive and negative emotion designs. Forty participants engaged in positive emotion tasks (twenty with high prosocial tendency and twenty with low prosocial tendency), and thirty-four participants engaged in negative emotion tasks (fourteen with high prosocial tendency and twenty with low prosocial tendency). The Greenhouse–Geisser freedom correction was used. All data were analyzed using SPSS 22.0.

### 2.5. Results

#### 2.5.1. Demographic Characteristics

Among the 74 healthcare students were 44 nursing students, 11 clinical medicine students, and 19 from other faculties. The demographic data between high prosocial and low prosocial groups are shown in Table 1.

#### 2.5.2. Emotional Contagion Task

In the negative emotional contagion task, the average response time was 2267.31 ms when the emotion expressed in the pictures was consistent with the words, and 2910.62 ms when they were inconsistent. The response time when the words were consistent with pictures to express negative emotion was significantly longer than when they were inconsistent (t = 4.325, 95% CI [351.577, 935.041], *p* < 0.001). Similarly, in the positive emotional contagion task, the average response time was 1641.68 ms when pictures and words had consistent emotions, and 3295.41 ms when they did not. The response time under consistent conditions was significantly shorter than that under inconsistent conditions (t = −10.691, 95% CI [−1957.155, −1350.319], *p* < 0.001).

#### 2.5.3. Outcomes of Prosocial Behavior Task

The results showed that the effects of a prosocial tendency (F = 9.141, 95% CI [1.889, 44.247], *p* = 0.006, η^2^ = 0.10) and emotional contagion (F = 6.453, 95% CI [1.282, 32.481], *p* = 0.024, η^2^ = 0.05) on the helping behavior of healthcare students were statistically significant (see Table 2). Furthermore, the interaction between the two factors was significant (F = 0.241, 95% CI [0.088, 0.659], *p* = 0.006, η^2^ = 0.11). A simple effect analysis showed statistical differences in the data of helping behavior between the two groups of low prosocial tendency students under different valences of emotional contagion (F = 7.842, *p* = 0.005, η^2^ = 0.21). There was no significant difference in healthcare students with high prosocial tendencies (*p* = 0.227). There was also no significant difference in the helping behavior of all students under different monetary rewards (*p* > 0.05).

## 3. Study 2

### 3.1. Participants

The participants of Study 2 are the same group as those in Study 1. To prevent interference between the two studies, we implemented a balanced experimental design: half of the students were randomly assigned to begin with Study 1, while the other half started with Study 2. This study excluded the nine participants who did not complete the help task, and one who had missing data. Finally, 40 participants with high prosocial tendencies and 41 participants with low tendencies were included in the analysis. However, to prevent interaction between studies, half were first randomly assigned to complete Study 1, and the other half first completed Study 2.

### 3.2. Materials

#### 3.2.1. Prosocial Tendency

The measurement scale was the same as Study 1.

#### 3.2.2. Epistemic Motivation Task

Fifty pictures were chosen from the Internet to serve as the task’s resources; 26 were related to the COVID-19 pandemic and 24 were unrelated. Then, we recruited 20 healthcare students to rate the valence and arousal of each picture on a 7-point scale, with points of 3–5 denoting neutral. Finally, we chose 32 pictures without emotional arousal to provide visual stimuli for epistemic motivation.

**Figure 3:**
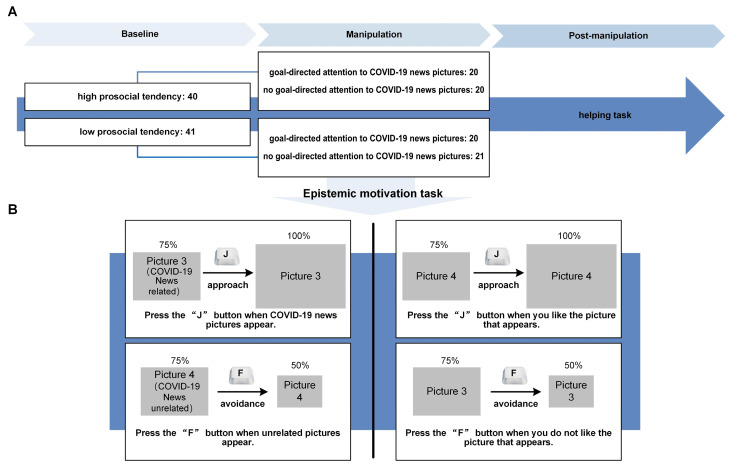
An Overview of the Procedure in Study 2. (**A**) Study 2 task path and (**B**) manipulations in the epistemic motivation task. All visual stimuli were displayed the same way as in Study 1. In the epistemic motivation task, participants were presented with 32 of the critical photographs randomly (16 COVID-19 news-related, 16 unrelated).

The manipulation of epistemic motivation was realized by the approach/avoid task [39,40]. All pictures were initially displayed to fill 75% of the screen. Participants in the goal-directed task were asked to press the “J” button to “approach” the COVID-19 picture when they saw it, causing the picture to enlarge to 100% of the screen within 3 s. Participants were then asked to visualize themselves approaching the scene as if it were real. In the no-goal-directed task, participants were asked to press the “J” button to “approach” the picture when they liked it, and to press the “F” button to “avoid” the picture when they did not like it (see Figure 3B).

#### 3.2.3. Prosocial Behavior Task

The task was the same as Study 1.

### 3.3. Procedure

Firstly, participants were asked to complete the epistemic motivation task. After this, the prosocial behavior task was given to them to finish. The experiments were conducted uniformly in the laboratory, and all participants signed an informed consent form before the experiment. A total of 30 CNY was given to each participant after the experiment.

### 3.4. Data Analysis

The study was modeled by a 2 (high prosocial tendency/low prosocial tendency) × 2 (goal-directed task/no-goal-directed task) mixed measure analysis of variance (ANOVA) (Figure 3A). According to the baseline prosocial tendency measurement, 81 participants were divided into a high prosocial tendency group (40 students) and a low prosocial tendency group (41 students). They were then randomly assigned to the intervention (goal-directed task) and control (no-goal-directed task) groups. The Greenhouse–Geisser freedom correction was used. All data were analyzed using SPSS 22.0.

### 3.5. Results

#### 3.5.1. Demographic Characteristics

Among the 81 healthcare students were 47 nursing students, 14 clinical medicine students, and 20 from other faculties. The demographic data of high prosocial and low prosocial groups are shown in Table 3.

#### 3.5.2. Epistemic Motivation Task

The response time of students in the goal-directed attention group to COVID-19 news pictures (mean 912.07 ms) was significantly shorter than that of other pictures (mean 1220.55 ms) (t = −4.439, 95% CI [−444.797, −172.173], *p* < 0.001). There was no significant difference in the response time to different pictures in the no-goal-directed attention group (*p* = 0.109).

#### 3.5.3. Outcomes of Prosocial Behavior Task

The results showed that the effects of prosocial tendencies on healthcare students’ helping behavior were significant (F = 0.126, 95% CI [0.022, 0.736], *p* = 0.021, η^2^ = 0.13). However, epistemic motivation had no significant effect on prosocial behaviors (*p* = 0.119). The interaction between the two factors was significant (F = 3.529, 95% CI [1.197, 10.401], *p* = 0.022, η^2^ = 0.13) (see Table 4). A simple effect analysis showed statistical differences in the data of helping behavior between the two groups of low prosocial tendency students, with or without goal-directed attention (F = 9.159, *p* = 0.003, η^2^ = 0.12). There was no significant difference in the high prosocial tendencies group (*p* = 0.848). There was also no significant difference in the helping behavior of all students under different monetary rewards (*p* > 0.05).

## 4. Discussion

This research aimed to investigate the effects of online information related to the pandemic on prosocial behavior among healthcare students. Through the use of an emotional contagion task, Study 1 indicated that healthcare students with low prosocial tendencies were more likely to decide not to perform prosocial behaviors when receiving negative COVID-19 information. Through the use of an epistemic motivation task, Study 2 showed that the low prosocial tendencies group was more likely to make helping decisions under the condition of no-goal-directed attention to the pictures of the pandemic. However, prosocial behavior in healthcare students with high prosocial tendencies is consistent and unaffected by the control of emotional contagion or epistemic motivation.

The results in Study 1 showed that students with poor prosocial tendencies were more likely to be discouraged from being helpful by negative information. Previous reports have indicated that the COVID-19 pandemic has been linked to mental health issues, such as depression and anxiety, which also affect students studying medicine [8,9,41]. These negative emotions would exacerbate ego depletion in people with low prosocial tendencies, and more ego depletion may cause fewer prosocial behaviors [2,42]. Conversely, positive emotion favors prosocial intention [7]. This is why even individuals with low prosocial tendencies are more likely to engage in helping behaviors in the face of positive pandemic information.

Prosocial activities are reportedly widespread during times of social trauma or crises [43]. However, individuals who were heavily exposed to news about major public health emergencies like COVID-19 showed fewer prosocial behaviors because of emotional exhaustion [43,44]. The results of Study 2 suggested that this phenomenon only happened in healthcare students with low prosocial tendencies. These results may relate to two reasons. Firstly, people with high prosocial tendencies had solid empathic abilities and were less easily influenced by their environment [45]; thus, even after learning more about the public health crises, their prosocial willingness remained persistent. Although public health emergencies might increase low prosocial peoples’ capacity for empathy and motivate them to engage in prosocial activities, excessive information impaired their ability to modulate their empathy and diminished their helping behaviors [21,46]. Secondly, individuals with strong prosocial impulses typically behave less egocentrically [47]. However, threats imposed by COVID-19 increase burnout and egocentric thinking in students with low prosocial tendencies, especially those participating in clinical fieldwork [1]. These findings align with previous research indicating that consuming COVID-19 information through social media and news negatively impacts the mental well-being of individuals with a stronger self-focus [48,49]. In fact, due to its limited medical resources and dense population, the Internet has been flooded with news concerning pandemic response and containment since the novel coronavirus outbreak. Thus, rational allocation of media resources to avoid broadcasting COVID-19 excessively might safeguard healthcare students’ desire to serve others.

Further, the prosocial behavior of healthcare students with high prosocial tendencies was less affected by online COVID-19 information, which was consistent with our previous hypothesis. High prosocial tendencies are associated with more cognitive emotional stability [30,50], which may be the primary factor explaining why these students are less impacted by pandemic information. Moreover, when people are socially conscious and compassionate, their prosocial activities are more driven by their moral principles, and less influenced by their environment [22,29,30]. Thus, this is why the helping behaviors of students with high prosocial tendencies were not influenced by pandemic information. However, inconsistent with our hypothesis, emotional contagion also did not affect the helping behaviors of students with high prosocial tendencies. Individuals with high prosocial tendencies often derive happiness and a sense of achievement due to their prosocial activity [51,52], which causes them to focus on prosocial action, rather than being influenced by external emotional information. It can be seen that it is important for healthcare students to cultivate their sense of responsibility, moral outlook, and other aspects to promote prosocial behaviors.

## 5. Limitations and Future Direction

Several limitations need to be considered. Firstly, the conclusions of this study are only appropriate for first- and second-year students without clinical experience. Senior students were excluded from our study because their extensive clinical experience in China may greatly influence their understanding of pandemic-related information and their participation in prosocial behaviors. Therefore, future research should focus on the factors influencing the prosocial behavior of senior healthcare students. Secondly, our participants were from the same college; thus, we recommend using stratified sampling in future research to obtain a more diverse sample of healthcare students from various institutions. Thirdly, we employed a self-report prosocial behavior measurement, which may be susceptible to individuals’ subjective attitudes. More objective measures or third-party behavior evaluations might reduce the potential for social desirability effects.

Furthermore, our results also provide further research directions. For one thing, the prosocial behaviors of healthcare providers were more affected by the pandemic than the healthcare students [2,53], and it is also meaningful to understand the specific reasons for the impact of the pandemic on their prosocial behaviors in future studies. For another, the COVID-19 pandemic has persisted for an extended period, with each stage influencing individuals’ attitudes and behaviors differently. Therefore, it is essential to conduct longitudinal tracking of these behavioral changes moving forward.

## 6. Conclusions and Implications

This research explored how online information related to the pandemic affects the prosocial behavior of healthcare students, and it presented enlightening findings. In summary, the prosocial behavior of healthcare students with low prosocial tendencies was more affected by pandemic information. On one hand, these students were less likely to engage in prosocial behavior after being influenced by negative information; on the other hand, when they focused excessively on information related to the pandemic (whether positive or negative), their likelihood of helping others decreased. Positive information guidance and moderate information exposure may, in part, boost their propensity to help others. Further, prosocial behavior in healthcare students with high prosocial tendencies is consistent and unaffected by the control of emotional contagion or epistemic motivation. Therefore, it is essential to help them learn to manage the responsibilities and pressures that come from work overload.

Our findings highlight an important gap in understanding how online pandemic information affects healthcare students’ prosocial behavior. The greatest insight from the research is that the emotional and behavioral changes of healthcare students, even those who were not directly involved in the pandemic, are worth paying attention to. In the context of the social pandemic, focusing on the causes and solutions of healthcare students’ prosocial behavior changes will guarantee high-quality public health services in the future. On one hand, some psychological support services that foster adaptive coping could be offered to groups of students broadly, across campuses. Additionally, avoiding excessive focus on negative pandemic-related information may assist healthcare students in mitigating their adverse emotional responses. On the other hand, enhancing healthcare students’ capacity to react to public health incidents and teaching them how to handle pandemics will assist them and lower the probability of negative emotions, thereby ensuring that their prosocial behaviors remain intact. Further, although high prosocial tendencies may benefit society, they may not always benefit the individuals themselves. For example, research has found that highly prosocial individuals may take on too much work, leading to stress, work overload, and reduced performance [54]. Therefore, targeted mental health education and guidance should be provided based on healthcare students’ unique psychological and behavioral traits.

## Figures and Tables

**Table 1:** Sociodemographic characteristics of students in high prosocial and low prosocial groups.

Characteristics	High Prosocial Group (n = 34)	Low Prosocial Group (n = 40)	*p*-Value
Age, year M (P_25_, P_75_)	19.5 (18.75, 20)	19 (19, 20)	0.413
Gender (n, %)			0.563
Male	15 (44.1)	15 (37.5)	
Female	19 (55.9)	25 (62.5)	
Grade (n, %)			0.420
Freshman	23 (67.6)	30 (75)	
Sophomore	10 (29.4)	7 (17.5)	
Junior and above	1 (2.9)	3 (7.5)	
Major (n, %)			0.119
Nursing	24 (70.6)	20 (50)	
Clinical medicine	5 (14.7)	6 (15)	
Other faculties *	5 (14.7)	14 (35)	
Perceived empathy M (P_25_, P_75_)	8 (8, 9)	7 (6.25, 8)	<0.001
Personal input M (P_25_, P_75_)	8 (7, 8)	7 (5, 7.75)	0.004
Prosocial tendencies M (P_25_, P_75_)	89 (85, 91.25)	69 (67, 72)	<0.001

Notes: Qualitative variables are expressed with absolute frequency (percentage), while quantitative variables are expressed with median (P_25_, P_75_). Perceived empathy and personal input were rated from 1–10 and self-reported by participants. * Other faculties in the high prosocial group are as follows: preventive medicine 2, forensic medicine 1, clinical pharmacy 1, rehabilitation medicine 1; other faculties in the low prosocial group are as follows: preclinical medicine 5, clinical pharmacy 2, rehabilitation medicine 1, preventive medicine 1, pediatrics 1, biomedical engineering 1, biostatistics 1, forensic medicine 1, radiation medicine 1.

**Table 2:** ANOVA on the effects of emotional contagion and tendencies on prosocial behaviors.

Variable	F	*p*-Value	Exp(B) 95%CI	
			Lower Limit	Upper Limit
Prosocial tendency	9.141	0.006	1.889	44.247
Emotional contagion	6.453	0.024	1.282	32.481
Prosocial tendency × Emotional contagion	0.241	0.006	0.088	0.659

**Table 3:** Sociodemographic characteristics of students in high prosocial and low prosocial groups.

Characteristics	High Prosocial Group (n = 40)	Low Prosocial Group (n = 41)	*p*-Value
Age, year M (P_25_, P_75_)	19 (19, 20)	19 (19, 20)	0.323
Gender (n, %)			0.586
Male	18 (45)	16 (39)	
Female	22 (55)	25 (61)	
Grade (n, %)			0.279
Freshman	27 (67.5)	31 (75.6)	
Sophomore	12 (30)	7 (17.1)	
Junior and above	1 (2.5)	3 (7.3)	
Major (n, %)			0.042
Nursing	27 (67.5)	20 (48.8)	
Clinical medicine	8 (20)	6 (14.6)	
Other faculties *	5 (12.5)	15 (36.6)	
Perceived empathy M (P_25_, P_75_)	8 (8, 9)	7 (6, 8)	<0.001
Personal input M (P_25_, P_75_)	8 (7, 8)	7 (5, 7.5)	0.003
Prosocial tendencies M (P_25_, P_75_)	89 (85.25, 91)	69 (67, 72)	<0.001

Notes: Qualitative variables are expressed with absolute frequency (percentage), while quantitative variables are expressed with median (P_25_, P_75_). Perceived empathy and personal input were from rated 1–10 and self-reported by participants. * Other faculties in the high prosocial group are as follows: preventive medicine 2, forensic medicine 1, clinical pharmacy 1, rehabilitation medicine 1; other faculties in the low prosocial group are as follows: preclinical medicine 5, rehabilitation medicine 2, clinical pharmacy 2, preventive medicine 1, pediatrics 1, biomedical engineering 1, biostatistics 1, forensic medicine 1, radiation medicine 1.

**Table 4:** ANOVA on the effects of epistemic motivation and tendencies on prosocial behaviors.

Variable	F	*p*-Value	Exp(B) 95%CI	
			Lower Limit	Upper Limit
Prosocial tendency	0.126	0.021	0.022	0.736
Epistemic motivation	0.264	0.119	0.049	1.410
Prosocial tendency × Epistemic motivation	3.529	0.022	1.197	10.401

## Data Availability

Analytic data of the present study are available at https://doi.org/10.6084/m9.figshare.24329632.v1.

## Abbreviations

The following abbreviations are used in this manuscript:

EASI	Emotions as a social information model
OR	Odds ratios
CI	Confidence intervals

## References

[1] Varma M.M., Chen D., Lin X., Aknin L.B., Hu X. (2023). Prosocial behavior promotes positive emotion during the COVID-19 pandemic. Emotion.

[2] Caldas M.P., Ostermeier K., Cooper D. (2021). When helping hurts: COVID-19 critical incident involvement and resource depletion in health care workers. J. Appl. Psychol..

[3] Zis P., Artemiadis A., Bargiotas P., Nteveros A., Hadjigeorgiou G.M. (2021). Medical Studies during the COVID-19 Pandemic: The Impact of Digital Learning on Medical Students’ Burnout and Mental Health. Int. J. Environ. Res. Public Health.

[4] Michno D.A., Tan J., Adelekan A., Konczalik W., Woollard A.C.S. (2021). How can we help? Medical students’ views on their role in the COVID-19 pandemic. J. Public Health.

[5] Tran V., Pham D.T., Dao T.N.P., Pham K.A.T., Ngo P.T., Dewey R.S. (2022). Willingness of Healthcare Students in Vietnam to Volunteer During the COVID-19 Pandemic. J. Community Health.

[6] Seah B., Ho B., Liaw S.Y., Ang E.N.K., Lau S.T. (2021). To Volunteer or Not? Perspectives towards Pre-Registered Nursing Students Volunteering Frontline during COVID-19 Pandemic to Ease Healthcare Workforce: A Qualitative Study. Int. J. Environ. Res. Public Health.

[7] Li L., Liu H., Wang G., Chen Y., Huang L. (2022). The Relationship Between Ego Depletion and Prosocial Behavior of College Students During the COVID-19 Pandemic: The Role of Social Self-Efficacy and Personal Belief in a Just World. Front. Psychol..

[8] Mamani B.S., Arias Y.M.A. (2023). Infodemic and Stress among Nursing Students in the Context of the COVID-19 Pandemic. Rev. Cuba. Enfermería.

[9] Ataoğlu B., Hıdıroğlu S., Çetin H., Gümüş E.G., Kılınç M., Köksal C., Karavuş M. (2023). A descriptive study assessing the infodemic medical students faced during COVID-19 pandemic. Eur. J. Public Health.

[10] Penner L.A., Dovidio J.F., Piliavin J.A., Schroeder D.A. (2005). Prosocial behavior: Multilevel perspectives. Annu. Rev. Psychol..

[11] Zhang J., Yin Y., Dean J., Zhang X., Zhang Y., Wang J., Zhang Y. (2021). Knowledge, Attitude, and Practice Survey of COVID-19 Among Healthcare Students During the COVID-19 Outbreak in China: An Online Cross-Sectional Survey. Front. Public Health.

[12] Van Kleef G.A., Homan A.C., Beersma B., van Knippenberg D. (2010). On angry leaders and agreeable followers. How leaders’ emotions and followers’ personalities shape motivation and team performance. Psychol. Sci..

[13] Van Kleef G.A., van den Berg H., Heerdink M.W. (2015). The persuasive power of emotions: Effects of emotional expressions on attitude formation and change. J. Appl. Psychol..

[14] Van Kleef G.A., Homan A.C., Beersma B., Van Knippenberg D., Van Knippenberg B., Damen F. (2009). Searing sentiment or cold calculation? The effects of leader emotional displays on team performance depend on follower epistemic motivation. Acad. Manag. J..

[15] Kramer A.D.I., Guillory J.E., Hancock J.T. (2014). Experimental evidence of massive-scale emotional contagion through social networks. Proc. Natl. Acad. Sci. USA.

[16] Stieglitz S., Dang-Xuan L. (2013). Emotions and information diffusion in social media—Sentiment of microblogs and sharing behavior. J. Manag. Inform. Syst..

[17] Balconi M., Canavesio Y. (2013). Prosocial attitudes and empathic behavior in emotional positive versus negative situations: Brain response (ERPs) and source localization (LORETA) analysis. Cogn. Process..

[18] Sweijen S.W., van de Groep S., Green K.H., Te Brinke L.W., Buijzen M., de Leeuw R.N.H., Crone E.A. (2022). Daily prosocial actions during the COVID-19 pandemic contribute to giving behavior in adolescence. Sci. Rep..

[19] Albert J., López-Martín S., Carretié L. (2010). Emotional context modulates response inhibition: Neural and behavioral data. NeuroImage.

[20] Wakslak C.J., Jost J.T., Tyler T.R., Chen E.S. (2007). Moral outrage mediates the dampening effect of system justification on support for redistributive social policies. Psychol. Sci..

[21] Noltemeyer A., Ward R.M., Fischbein R., Bonfine N., Ritter C., Zierden C., Seok J. (2022). Health professions student helping behaviors and attitudes toward a person experiencing anxiety within the context of COVID-19. Int. J. Ment. Health.

[22] Jiang Y., Yao Y., Zhu X., Wang S. (2021). The Influence of College Students’ Empathy on Prosocial Behavior in the COVID-19 Pandemic: The Mediating Role of Social Responsibility. Front. Psychiatry.

[23] Todd A.R., Forstmann M., Burgmer P., Brooks A.W., Galinsky A.D. (2015). Anxious and egocentric: How specific emotions influence perspective taking. J. Exp. Psychol. Gen..

[24] Batson C.D., Powell A.A. (2003). Altruism and Prosocial Behavior. Handbook of psychology: Personality and social psychology.

[25] Rokhim R., Devina M. (2019). Contact employees’ prosocial behaviors: The role of leader-member exchange and perceived organizational support. Leading for High Performance in Asia: Contemporary Research and Evidence-Based Practices.

[26] Kim H., Qu H. (2020). The mediating roles of gratitude and obligation to link employees’ social exchange relationships and prosocial behavior. Int. J. Contemp. Hosp. Manag..

[27] Negd M., Mallan K.M., Lipp O.V. (2011). The role of anxiety and perspective-taking strategy on affective empathic responses. Behav. Res. Ther..

[28] Schroeder D.A., Graziano W.G. (2015). The Oxford Handbook of Prosocial Behavior.

[29] Grant A.M. (2008). Does intrinsic motivation fuel the prosocial fire? Motivational synergy in predicting persistence, performance, and productivity. J. Appl. Psychol..

[30] Habashi M.M., Graziano W.G., Hoover A.E. (2016). Searching for the Prosocial Personality: A Big Five Approach to Linking Personality and Prosocial Behavior. Pers. Soc. Psychol. B.

[31] Dai H., Zhang F. (2018). Psychological & Educational Measurement.

[32] Carlo G., Randall B.A. (2002). The development of a measure of prosocial behaviors for late adolescents. J. Youth Adolesc..

[33] Kou Y., Hong H.F., Tan C., Li L. (2007). Revisioning prosocial tendencies measure for adolescent. Psychol. Dev. Educ..

[34] Watson D., Clark L.A., Tellegen A. (1988). Development and validation of brief measures of positive and negative affect: The PANAS scales. J. Pers. Soc. Psychol..

[35] Lang P.J., Bradley M.M., Cuthbert B.N. (1999). International Affective Picture System (IAPS): Instruction Manual and Affective Ratings.

[36] Ji L.L., Peng H.M. (2016). The Role of Emotional Information and Internal Motivation in Older Adults’ Decision-making Process. Prog. Biochem. Biophys..

[37] Fazio R.H., Sanbonmatsu D.M., Powell M.C., Kardes F.R. (1986). On the automatic activation of attitudes. J. Pers. Soc. Psychol..

[38] Gross J., Faber N.S., Kappes A., Nussberger A.M., Cowen P.J., Browning M., Kahane G., Savulescu J., Crockett M.J., De Dreu C.K.W. (2021). When Helping Is Risky: The Behavioral and Neurobiological Trade-off of Social and Risk Preferences. Psychol. Sci..

[39] Cunningham W.A., Arbuckle N.L., Jahn A., Mowrer S.M., Abduljalil A.M. (2011). Reprint of: Aspects of neuroticism and the amygdala: Chronic tuning from motivational styles. Neuropsychologia.

[40] Deng Y., Li S., Zhou R., Walter M. (2018). Motivation but not valence modulates neuroticism-dependent cingulate cortex and insula activity. Hum. Brain. Mapp..

[41] Rajkumar R.P. (2020). COVID-19 and mental health: A review of the existing literature. Asian J. Psychiatr..

[42] Osgood J.M., Muraven M. (2015). Self-control depletion does not diminish attitudes about being prosocial but does diminish prosocial behaviors. Basic Appl. Soc. Psychol..

[43] Zaki J. (2020). Catastrophe Compassion: Understanding and Extending Prosociality Under Crisis. Trends Cogn. Sci..

[44] Huckins J.F., daSilva A.W., Wang W., Hedlund E., Rogers C., Nepal S.K., Wu J., Obuchi M., Murphy E.I., Meyer M.L. (2020). Mental Health and Behavior of College Students During the Early Phases of the COVID-19 Pandemic: Longitudinal Smartphone and Ecological Momentary Assessment Study. J. Med. Internet Res..

[45] Davis M.H., Franzoi S.L. (1991). Stability and change in adolescent self-consciousness and empathy. J. Res. Pers..

[46] Padilla-Walker L.M., Coyne S.M., Collier K.M., Nielson M.G. (2015). Longitudinal relations between prosocial television content and adolescents’ prosocial and aggressive behavior: The mediating role of empathic concern and self-regulation. Dev. Psychol..

[47] Li T., Siu P.M. (2021). Socioeconomic Status Moderates Age Differences in Empathic Concern. J. Gerontol. B-Psychol..

[48] Bu F., Steptoe A., Fancourt D. (2020). Loneliness during lockdown: Trajectories and predictors during the COVID-19 pandemic in 35,712 adults in the UK. medRxiv.

[49] Gao J., Zheng P., Jia Y., Chen H., Mao Y., Chen S., Wang Y., Fu H., Dai J. (2020). Mental health problems and social media exposure during COVID-19 outbreak. PLoS ONE.

[50] Rahal R.M., Fiedler S. (2022). Cognitive and affective processes of prosociality. Curr. Opin. Psychol..

[51] Sin N.L., Klaiber P., Wen J.H., DeLongis A. (2021). Helping Amid the Pandemic: Daily Affective and Social Implications of COVID-19-Related Prosocial Activities. Gerontologist.

[52] Weinstein N., Ryan R.M. (2010). When helping helps: Autonomous motivation for prosocial behavior and its influence on well-being for the helper and recipient. J. Pers. Soc. Psychol..

[53] Bazán P.R., de Azevedo Neto R.M., Lacerda S.S., Ribeiro M.W., Balardin J.B., Amaro E., Kozasa E.H. (2021). Can news with positive or negative content affect and a relaxation pause improve the emotional state of health care professionals? A randomized online experiment during COVID-19 pandemic. Internet Interv..

[54] Kibler E., Wincent J., Kautonen T., Cacciotti G., Obschonka M. (2019). Can prosocial motivation harm entrepreneurs’ subjective well-being?. J. Bus. Ventur..

